# Structural basis for enzymatic terminal C–H bond functionalization of alkanes

**DOI:** 10.1038/s41594-023-00958-0

**Published:** 2023-03-30

**Authors:** Jin Chai, Gongrui Guo, Sean M. McSweeney, John Shanklin, Qun Liu

**Affiliations:** 1 Biology Department, Brookhaven National Laboratory, Upton, NY USA; 2 NSLS-II, Brookhaven National Laboratory, Upton, NY USA

**Keywords:** Cryoelectron microscopy, Oxidoreductases

## Abstract

Alkane monooxygenase (AlkB) is a widely occurring integral membrane metalloenzyme that catalyzes the initial step in the functionalization of recalcitrant alkanes with high terminal selectivity. AlkB enables diverse microorganisms to use alkanes as their sole carbon and energy source. Here we present the 48.6-kDa cryo‐electron microscopy structure of a natural fusion from *Fontimonas thermophila* between AlkB and its electron donor AlkG at 2.76 Å resolution. The AlkB portion contains six transmembrane helices with an alkane entry tunnel within its transmembrane domain. A dodecane substrate is oriented by hydrophobic tunnel-lining residues to present a terminal C–H bond toward a diiron active site. AlkG, an [Fe–4S] rubredoxin, docks via electrostatic interactions and sequentially transfers electrons to the diiron center. The archetypal structural complex presented reveals the basis for terminal C–H selectivity and functionalization within this broadly distributed evolutionary class of enzymes.

## Abstract (web summary)

Alkane monooxygenase (AlkB) enables diverse microorganisms to hydrolyze alkanes as their sole carbon and energy source. Liu and colleagues reveal how AlkB orients an alkane to its active site and performs a selective terminal C–H bond hydroxylation.

## Main

Alkanes are saturated hydrocarbons that constitute 20–50% of crude oils. Due to their abundance and low cost, alkanes are attractive starting materials for producing chemical feedstocks and value-added products^1,2^. Alkanes contain hydrocarbons with fully occupied orbitals for their C–H bonds and similar bond energies, rendering them almost chemically inert and challenging to functionalize selectively. Alkane monooxygenase (AlkB) is a transmembrane metalloenzyme that catalyzes terminal hydroxylation reactions of alkanes with broad chain-length specificity and exclusive terminal C–H bond selectivity^3,4^. AlkB can be engineered to allow it to derivatize abundant alkanes to alcohols, aldehydes, carboxylic acids and epoxides^5,6^.

**Extended Data Fig. 1:**
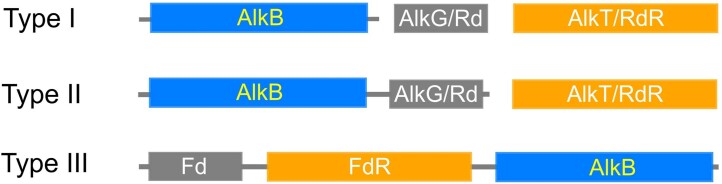
Organization of the components of the AlkBGT biocatalysts. The three components (AlkB, AlkG, and AlkT) in the alkane hydroxylation biocatalyst are encoded as separate genes in *P. oleovorans*. AlkG is an Fe-sulfur cluster-containing redox protein that transfers electrons to the diiron center in AlkB. AlkT is a flavin adenine dinucleotide (FAD)-dependent rubredoxin reductase that transfers two electrons from NADH singly to the Fe-sulfur cluster of AlkG, which then relays two of them singly to AlkB to enable biocatalysis^10,12^. The membrane-bound AlkB is a nonheme diiron-containing monooxygenase that specifically oxidizes the terminal methyl group of alkanes to produce the corresponding primary alcohols^8,10,13,14,17,22,30^. There are microbes that have evolved to have the AlkB and AlkG fused together, presumably for improved electron transfer^15,26^. Additionally, in some species (Leptospira, Limnobacter, and Polaromonas), AlkB is fused with multiples proteins of an N-terminal ferredoxin and a ferredoxin reductase^15^.

AlkB was originally identified in *Pseudomonas oleovorans*, which thrives in oil-rich environments where it uses alkanes as its sole carbon and energy source^7–10^. The first and key step in alkane utilization is its hydroxylation, which requires an electron transfer and redox system of AlkBGT, consisting of a catalytic membrane-bound monooxygenase AlkB, a soluble rubredoxin AlkG and a soluble rubredoxin reductase AlkT^11,12^. AlkB, AlkG and AlkT can be encoded as separate or fusion proteins^13–15^ (Extended Data Fig. 1). Intense research over the past half century has been devoted to characterizing and engineering the AlkBGT system for alkane C–H functionalization^10,16–21^. The structure of this electron transfer complex and the molecular determinants of alkane terminal selectivity have nonetheless remained elusive.

## Results

### Structure determination

**Extended Data Fig. 2:**
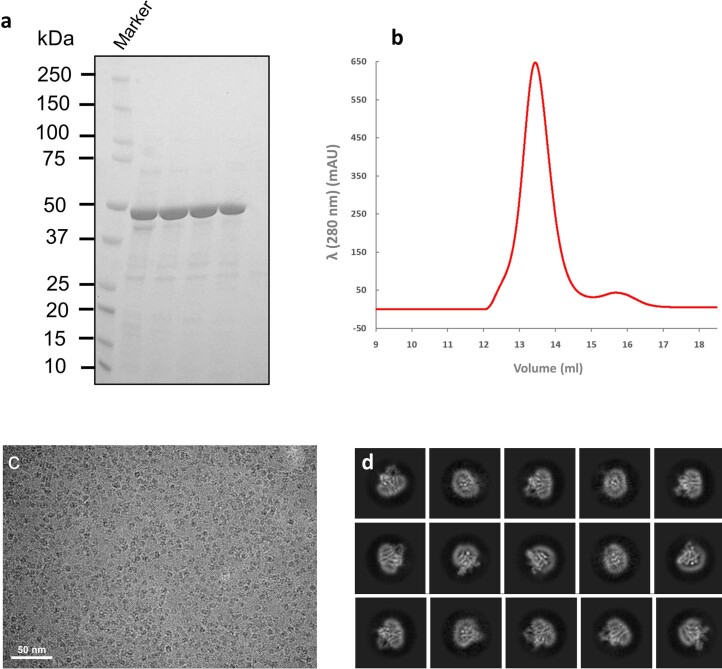
Production and analysis of reconstituted *Ft*AlkBG particles. **a**, SDS-PAGE gel analysis of purified *Ft*AlkBG in detergent DDM. This experiment was reproducible and was repeated for at least three times. **b**, Size-exclusion chromatography profile of *Ft*AlkBG reconstituted into PMAL-C8 nanodiscs. **c**, A typical motion-corrected cryo-EM micrograph. We have collected a total of 11,757 cryo-EM micrographs of similar participle size and distribution. **d**, 2D class averages showing secondary structure features of *Ft*AlkBG with its transmembrane region wrapped by PMAL-C8. Source data

We screened 21 AlkB homologs for production in *Escherichia coli* and identified a natural AlkB-AlkG fusion from *Fontimonas thermophila* (*Ft*AlkBG) that was expressed well and could be purified using the detergent *n*-dodecyl-β-d-maltoside (DDM) (Extended Data Fig. 2a). We reconstituted the protein in amphipol PMAL-C8 nanodiscs and further purified it using size-exclusion chromatography (Extended Data Fig. 2b). The molecular mass of *Ft*AlkBG on SDS-PAGE is less than 50 kDa (Extended Data Fig. 2a), which is relatively small for structure determination by cryo‐electron microscopy (cryo-EM). To enhance the signals of cryo-EM images, we used a camera pixel size of 0.333-Å resolution under super-resolution mode, with an energy filter slit width at 15 eV, and collected data from holes with the thinnest-possible ice capable of encapsulating particles. Under these conditions, we were able to obtain well-identifiable particles on micrographs (Extended Data Fig. 2c). Two-dimensional (2D) class averaging showed recognizable secondary structural features and amphipol densities around the protein transmembrane region (Extended Data Fig. 2d).

**Extended Data Fig. 3:**
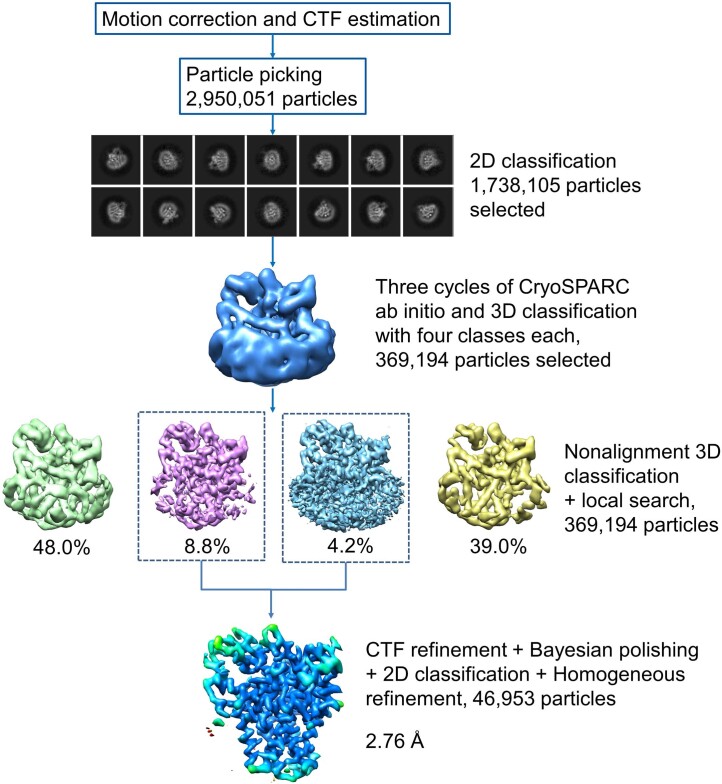
Flow chart of cryo-EM data processing. The flow chart includes motion correction and CTF estimation, 2D and 3D class averages, and 3D refinement.

**Extended Data Fig. 4:**
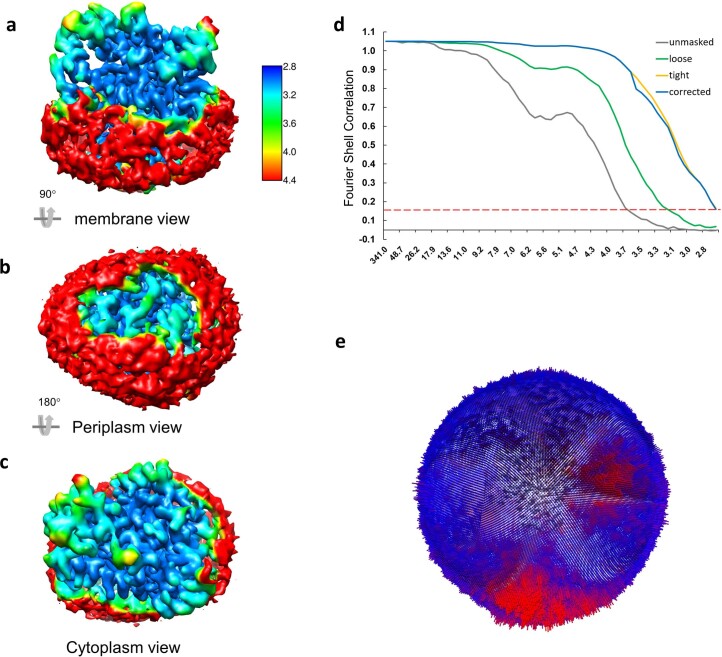
3D reconstruction of *Ft*AlkBG. **a-c**, Three views for the reconstructed map colored with local resolutions. Red belts are amphipol densities around the transmembrane regions of the complex. **d**, Fourier Shell Correlation (FSC) curves for the 3D reconstruction. **e**, Angular distribution for particles used in the final 3D reconstruction. Source data

**Extended Data Fig. 5:**
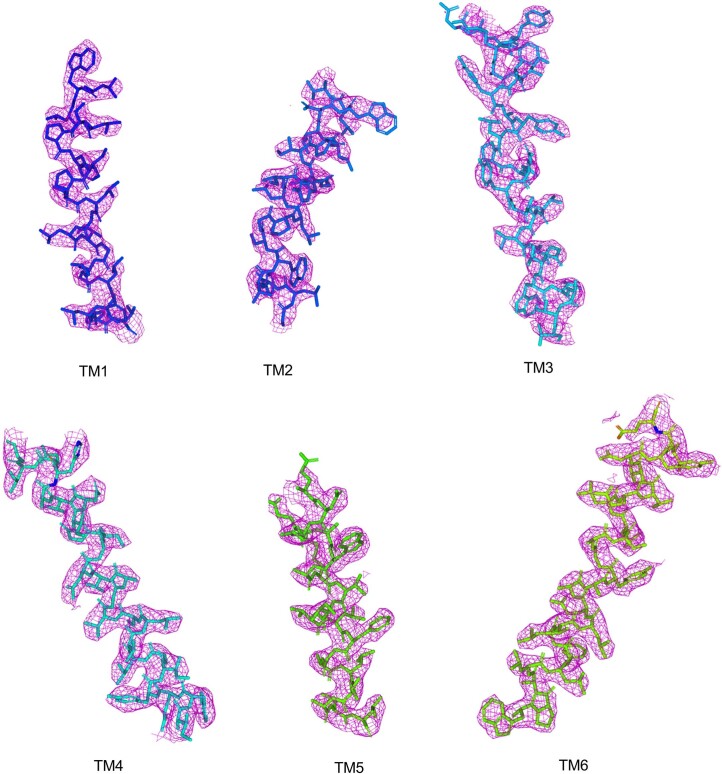
Quality of the cryo-EM maps. Cryo-EM densities for the six transmembrane regions (TM1-6) are shown as magenta isomeshes. The six transmembrane helices are shown as sticks.

We determined the structure of *Ft*AlkBG, using the workflow illustrated in Extended Data Fig. 3, and achieved a cryo-EM map at a resolution of 2.76 Å (Table 1). *Ft*AlkBG is wrapped tightly by amphipols that cover approximately half of the protein (Extended Data Fig. 4a–c). Amphipols appear to form a shield that stabilizes the transmembrane region of *Ft*AlkBG during sample vitrification. In addition, the relatively small size of the PMAL-C8 amphipol may be well suited for cryo-EM structure determination of small membrane proteins. The final reconstruction used only 1.6% (46,953) of the total picked particles to reach a resolution of 2.76 Å, estimated using the gold-standard Fourier shell correlation at 0.143 (Extended Data Fig. 4d). These particles have well-distributed orientation angles, with only slightly more particles viewed from the periplasmic side (Extended Data Fig. 4e). The cryo-EM map is of high quality and allowed the building and refinement of an atomic model (Extended Data Fig. 5).

**Table 1:** Cryo-EM data collection, 3D reconstruction and refinement statistics

	*Ft*AlkBG (EMD-28890, PDB 8F6T)
**Data collection**	
Microscope	Titan Krios G3i
Magnification	×130,000
Stage type	Autoloader
Voltage (kV)	300
Detector	Gatan K3
Energy filter (eV)	15
Acquisition mode	Super-resolution
Physical pixel size (Å)	0.666
Defocus range (µm)	0.7–2.5
Electron exposure (e^−^ Å^−^^2^)	60
**Reconstruction**	
Software	Relion v.3.08, CryoSPARC v.2.15
Particles picked (no.)	2,950,051
Particles final (no.)	46,953
Extraction box size (pixels)	128
Rescaled box size (pixels)	64
Final pixel size (Å)	1.332
Symmetry imposed	C1
Map resolution (Å)	2.76
FSC threshold	0.143
Map resolution range (Å)	341–2.72
Map sharpening *B* factor (Å^2^)	66
**Model refinement**	
Software	PHENIX
Refinement algorithm	Real Space
Clipped box size (pixels)	None
Residues (no.)	428
Iron (no.)	3
Ligand (no.)	1
**R.m.s.** **deviations**	
Bond lengths (Å)	0.004
Bond angles (°)	0.573
**Validation**	
MolProbity clashscore	4.79
Rotamer outliers (%)	0.0
Cβ deviations (%)	0.0
**Ramachandran plot**	
Favored (%)	94.58
Allowed (%)	5.42
Outliers (%)	0

### Overall structure

**Fig. 1:**
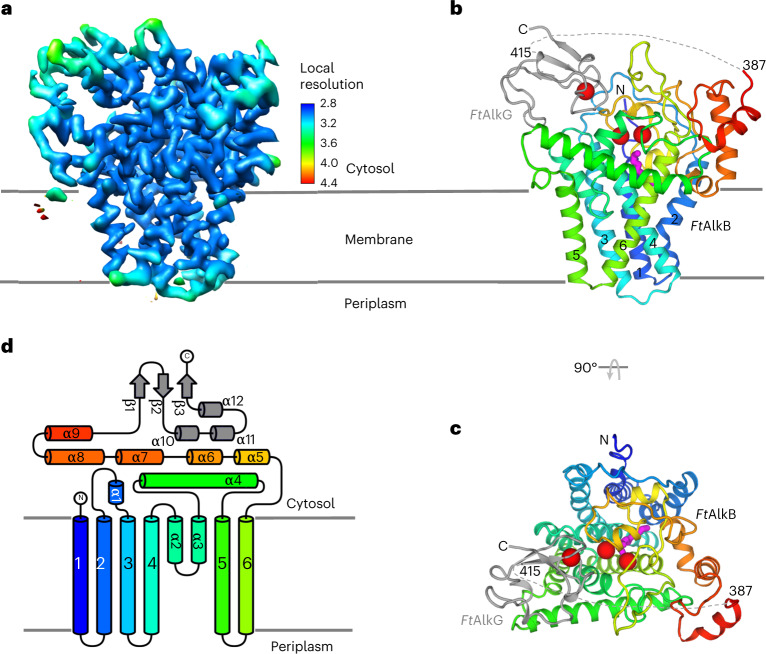
Overall structure. **a**, Cryo-EM density map colored with local resolutions. **b**,**c**, Two views of the overall structure: side view (**b**) and top view with a 90° rotation from **b** (**c**). *Ft*AlkB is shown as rainbow cartoons, with colors ranging from blue for N terminus to red for C terminus. *Ft*AlkG is shown in gray. Three irons, two in *Ft*AlkB and one in *Ft*AlkG, are shown as red spheres. The substrate dodecane is drawn as magenta sticks. The six transmembrane helices are labeled by numbers 1–6. **d**, Secondary structure topology of the structure. The coloring is the same as in **b**. Six transmembrane helices are indicated by numbers 1–6.

The solved *Ft*AlkBG structure is a monomer (Fig. 1a), consisting of both AlkB (residues 13–387) and AlkG (residues 415–467) domains (Fig. 1b,c), with a total molecular mass of 48.6 kDa. The *Ft*AlkB structure is mostly α-helical, with six transmembrane α-helices (TM1–6), two partially membrane-embedded short α-helices α2 and α3 (Fig. 1d), a long membrane-associated amphiphilic helix α4 (Fig. 1b,d) and six other α-helices outside the membrane (Fig. 1c,d). TM2 is kinked within the membrane, making an angle of about 100°. The protein is narrower on the periplasmic side than on the cytoplasmic side, forming a wedge-shaped structure that may be responsible for bending the membranes to facilitate the formation of membrane vesicles^22^. In terms of sequence, TM1–4 are clustered and separated from TM5 and TM6 by α2–α4. The soluble domain follows TM6 and connects to the *Ft*AlkG domain through a 27-residue, disordered linker (residues 388–414).

### The diiron active site

**Fig. 2:**
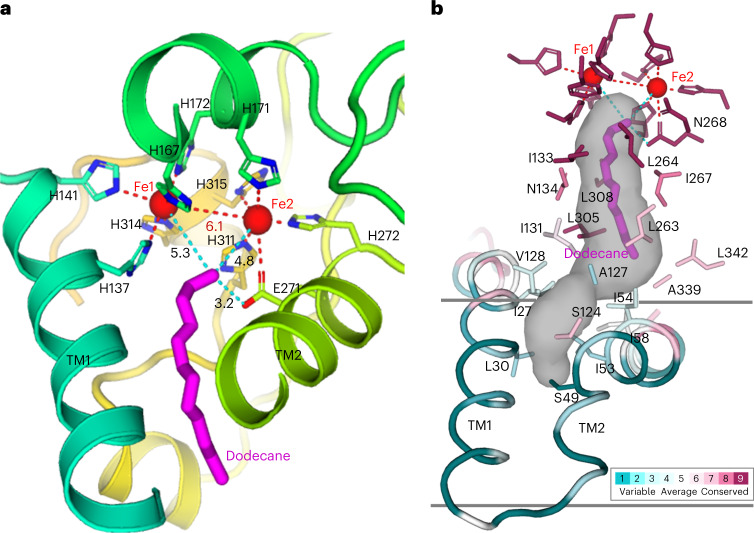
Active site structure. **a**, Structure of the diiron center with a substrate, dodecane, bound. The diiron center (Fe1 and Fe2, red spheres) interacts with nine histidines and one glutamate (sticks) and forms the diiron-center structure. Substrate is shown as magenta sticks, and its distance to the diiron and Glu271 are shown as dashed lines in cyan. **b**, Substrate binding site and substrate entry tunnel. Residues forming the tunnel are shown as sticks and colored by degree of conservation. The substrate, dodecane, is shown as magenta sticks. The entrance of the tunnel is in the membrane between TM1 and TM2.

**Extended Data Fig. 6:**
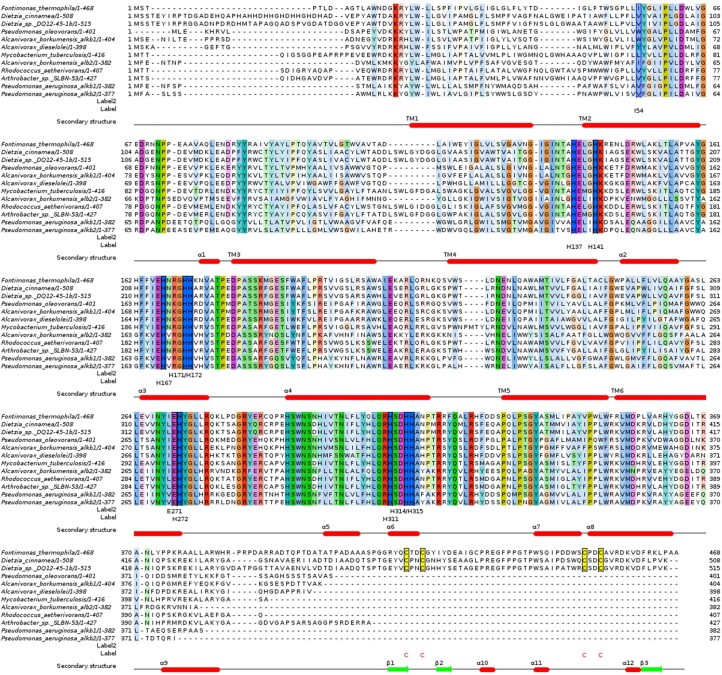
Structure-based sequence alignments of AlkBs. Nine conserved histidine and one glutamate residues are indicated. Residue I54 and its equivalent residues in other AlkBs are also indicated. Conserved cysteine residues in three AlkBG fusions are highlighted in yellow.

**Extended Data Fig. 7:**
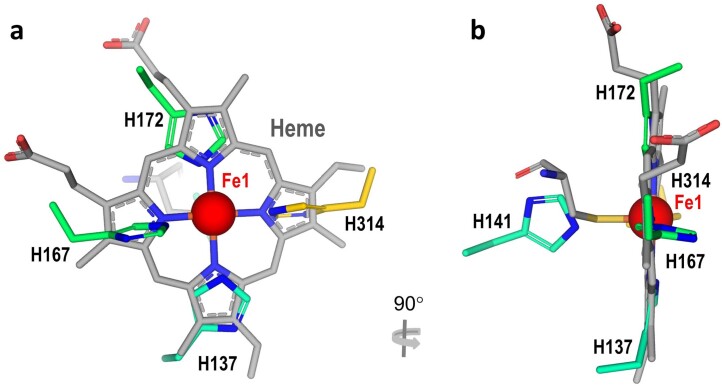
Superimposition of the Fe1 site with heme in cytochrome P450. **a**, The Fe1-center was superimposed with the heme (gray sticks) in a cytochrome P450 monooxygenase (PDB code 6H1T). The four nitrogen atoms on four histidines are in the same plane as the heme and align well with the four porphyrin inner nitrogen atoms. **b**, A view of 90° rotation of (**a**). His141 is behind the Fe1-hisitine plane to further stabilize Fe1.

*Ft*AlkB binds two iron ions, which form a diiron center that is essential for C–H bond activation (Fig. 2)^23^. The diiron center is coordinated by nine conserved histidines and one carboxylate residue (Extended Data Fig. 6) with His137, His141, His167, His172 and His314 coordinating Fe1 and four histidines (His171, His272, His311 and His315) and glutamate Glu271 coordinating Fe2. The coordination geometry of Fe1 is octahedral, such that four histidine nitrogen atoms are coplanar with Fe1, analogous to the heme in cytochrome P450 enzymes that can also catalyze C–H bond hydroxylation (Extended Data Fig. 7)^24^. The octahedral geometry of Fe1 allows a fifth ligand on one side of the plane. His141 is on the other side of the plane to further stabilize Fe1. Fe2 is 6.1 Å away from Fe1, in line with Fe1 and His141, but too far to coordinate with Fe1. The four histidines (His171, His272, His311 and His315) are on the same side of the Fe2 site, thus allowing additional coordination to Fe2.

**Extended Data Fig. 8:**
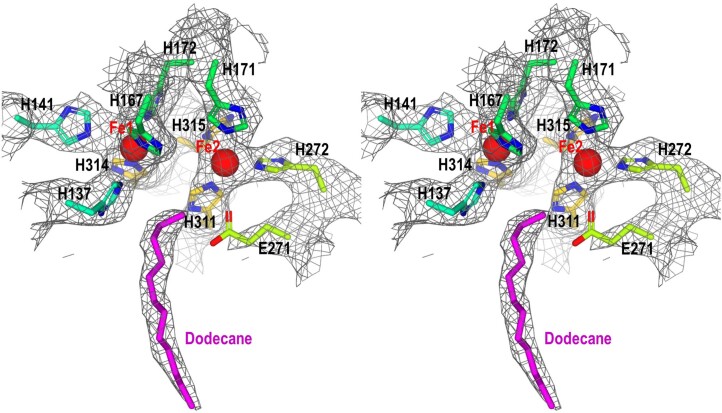
A stereo view of cryo-EM densities for the active site. The densities are shown as gray isomeshes covering two irons (red spheres), substrate (magenta sticks), nine histidines (sticks) and one glutamate (sticks).

**Extended Data Fig. 9:**
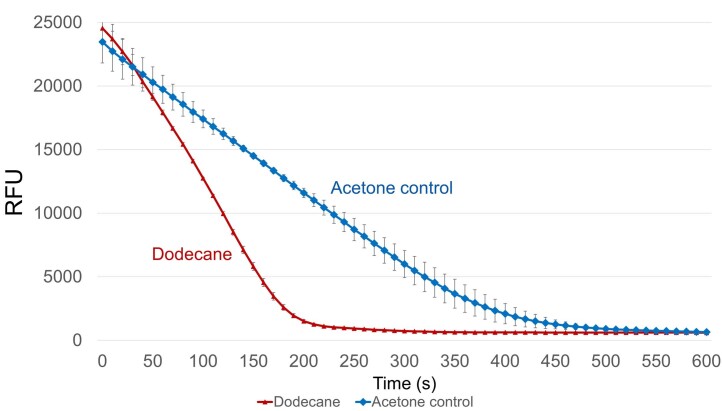
*Ft*AlkBG activity measured by NADH fluorescence intensity. The consumption of NADH was monitored by NADH fluorescence with ex/em of 360/460 nm using a plate reader. Conversion of dodecane to dodecanol resulted in the increased consumption of NADH, while the addition of acetone control decreased the NADH fluorescence intensity slowly. RFU is relative fluorescence unit. The error bar is the standard deviation (s.d.) of the mean from three independent experiments. Source data

Additional density was present in the active site of the cryo-EM structure, allowing a dodecane substrate (C-12) to be modeled with its terminal methyl group in close proximity of the diiron center (Fig. 2b and Extended Data Fig. 8). Experimentally, *Ft*AlkBG hydroxylates dodecane, as demonstrated by the decrease in NADH fluorescence compared to a nonsubstrate control (Extended Data Fig. 9). No alkanes were provided during protein production or sample preparation. Therefore, the dodecane was likely derived from the *E. coli* which was used as the expression host for *Ft*AlkBG. The linear dodecane is oriented by hydrophobic residues, such that its terminal methyl group (C(*sp*^3^)–H bonds) is 5.3 Å from Fe1 and 4.8 Å from Fe2. The methyl group is also located 3.2 Å away from Glu271, suggesting a possible role for Glu271 in hydroxylation of the C–H bond (Fig. 2a). The methyl group is not in line with Fe1 and His141. Instead, it is closer to Fe2 and lies on the noncoordinated side of Fe2. The proximity of the substrate terminal methyl group to Fe2 suggests the Fe2 center may be more directly involved in C–H bond activation.

### Substrate selectivity

*Ft*AlkBG has a long, hydrophobic substrate binding channel starting from near the middle of transmembrane helices TM1 and TM2 and ending at the diiron center (Fig. 2b). The hydrophobic residues lining the channel show increasing degrees of conservation the nearer they are to the diiron site. Dodecane occupies roughly half the tunnel length, suggesting the enzyme could accommodate longer alkanes. Because the tunnel entrance is narrower than the dodecane-occupied region, binding of longer substrates would presumably be accommodated by minor conformational changes. The side chains of residues Ile27, Ile54 and Ala127 form a restriction site. Sequence alignment shows that Ile54 in *Ft*AlkBG is equivalent to Trp55 in AlkB from *P. oleovorans* (*Po*AlkB) (Extended Data Fig. 6). Trp55 in *Po*AlkB determines alkane chain-length selectivity because mutating it to a serine or cysteine extended its alkane chain-length selectivity from C-10 to C-13 (ref. ^25^). Conversely, AlkB in *Mycobacterium tuberculosis* has a leucine at position 69 (equivalent to Trp55 in *Po*AlkB and Ile54 in *Ft*AlkBG); mutating it to a bulky side chain residue phenylalanine or tryptophan reduces the chain-length selectivity from C-16 to C-11 (ref. ^25^). Similarly, in AlkBG from *Dietzia cinnamea*, a V91W mutant (equivalent to Ile54 in *Ft*AlkBG) rendered the enzyme less active for alkanes longer than C-9 (ref. ^26^). Collectively, these hydrophobic residues appear to mediate alkane chain-length selectivity.

### Electron transfer mechanism

**Fig. 3:**
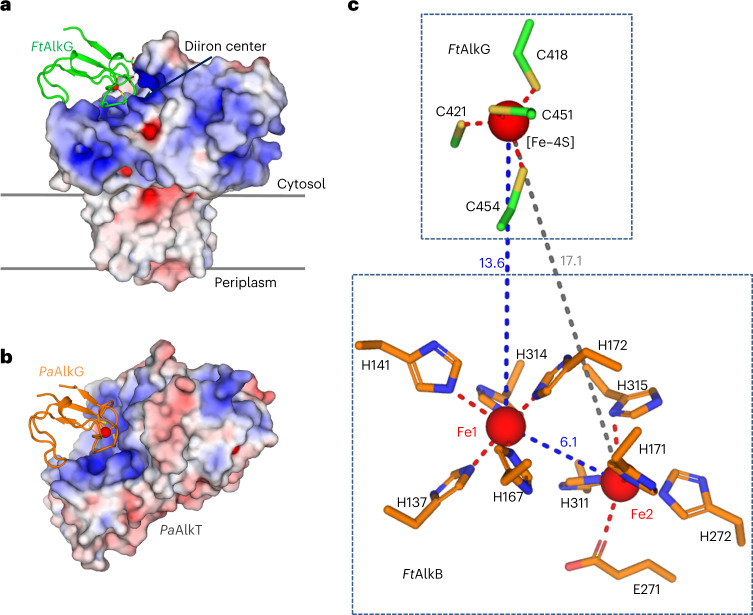
Electron transfer mechanism. **a**, Positioning of *Ft*AlkG relative to *Ft*AlkB for electron transfer. *Ft*AlkB is shown as electrostatic potential surface. The electrostatics was calculated using the program APBS^36^ and plotted at the level of ±5 *kT/e*. **b**, Positioning of AlkG relative to AlkT from *Pseudomonas aeruginosa* (*Pa*AlkT and *Pa*AlkG, respectively; PDB 2V3B)^27^. *Pa*AlkT is shown as electrostatic potential surface. *Pa*AlkG is shown as a ribbon with its iron center shown as a red sphere. The electrostatics was calculated using the program APBS and plotted at the level of ±5 *kT/e*. **c**, Geometry of electron transfer between *Ft*AlkG and *Ft*AlkB. The distances between the [Fe–4S] cluster to the two irons are indicated as blue and gray dashed lines. The distance between Fe1 and Fe2 is indicated as a blue dashed line. Residues forming the diiron center site are shown as sticks.

**Extended Data Fig. 10:**
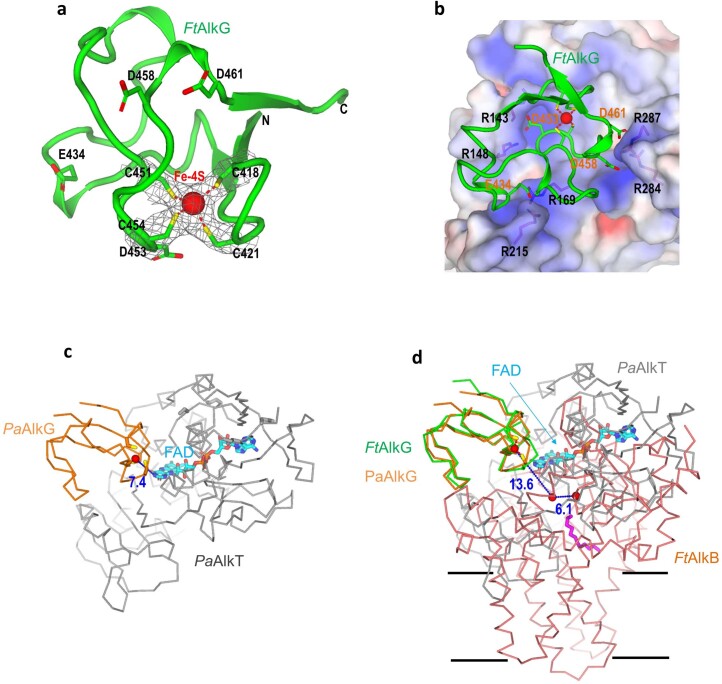
AlkG in electron transfer. **a**, *Ft*AlkG structure and its [Fe-4S] iron-sulfur cluster. The iron is shown as a red sphere and the four cysteine residues are shown as sticks. Four negatively charged residues are also shown as sticks. Cryo-EM density for the iron-sulfur cluster was shown as gray isomeshes. **b**, Electrostatic interactions between *Ft*AlkB and *Ft*AlkG. Positively charged arginine residues on AlkB and negatively charged glutamate or aspartate residues are shown as sticks. **c**, Electron transfer between *Pa*AlkT and *Pa*AlkG at a distance of 7.4 Å from FAD to the iron center. **d**, AlkG on and off from AlkT and AlkB for electron transfer. We superimposed the *Ft*AlkBG structure with *Pa*AlkG-AlkT complex. The sequence identity between *Ft*AlkG and *Pa*AlkG is 54.5%. The superimposition was performed on the *Ft*AlkG and *Pa*AlkG with an R.M.S.D. of 0.932 Å for 49 aligned Cα atoms. In the superimposed structure, AlkT and AlkB bind to the same AlkG site to transfer and receive an electron, respectively. *Ft*AlkB, salmon; *Ft*AlkG, green; *Pa*AlkT, gray; *Pa*AlkG, orange. FAD molecule in *Pa*AlkT is drawn as sticks. The three irons in the *Ft*AlkBG are shown as red spheres. Substrate dodecane is shown as magenta sticks.

C–H bond activation requires the transfer of electrons to the Fe–Fe diiron center in AlkB by AlkG^11^. *Ft*AlkG has an [Fe–4S] cluster coordinated by Cys418, Cys421, Cys451 and Cys454 (Extended Data Fig. 10a). *Ft*AlkG sits on a positively charged surface of *Ft*AlkB, adjacent to the diiron center (Fig. 3a). On the surface of AlkG are four negatively charged residues (that is, Glu434, Asp453, Asp458 and Asp461; Extended Data Fig. 10b) that form electrostatic interactions with six positively charged residues (Arg143, Arg148, Arg169, Arg215, Arg284 and Arg287) on the surface of *Ft*AlkB. However, we did not observe specific salt-bridge interactions between carboxylate–guanidinium pairs in the cryo-EM density. We interpret this to mean that nonspecific electrostatic interactions may facilitate fast on and off rates of *Ft*AlkG association with *Ft*AlkB that would act as a shuttle to relay pairs of electrons one at a time from *Ft*AlkT to *Ft*AlkB. This mechanism is also consistent with the disordering of the linker between *Ft*AlkG and *Ft*AlkB (Fig. 1b).

AlkG alone cannot supply electrons; it functions as a shuttle of electrons originating from its electron donor NADH through NADH oxidation by AlkT, a rubredoxin reductase^12^. Electron transfer between AlkT and AlkG has been proposed on the basis of the structure of a complex between rubredoxin reductase and rubredoxin from *P. aeruginosa* (*Pa*AlkT and *Pa*AlkG, respectively) (Extended Data Fig. 10c)^27^. We superimposed the *Ft*AlkBG and *Pa*AlkG-*Pa*AlkT structures on the basis of AlkG orientation (Extended Data Fig. 10d). We found that AlkB and AlkT bind to the same face on AlkG (Fig. 3a,b), indicating AlkG must dissociate from AlkB to associate with AlkT to enable its reduction and subsequently transfer the electron to AlkB, thus forming a sequential electron transfer model shuttling one electron at a time.

Although there are two irons in *Ft*AlkB, the [Fe–4S] cluster in *Ft*AlkG is closer to Fe1, that is, at 13.6 Å, than to Fe2, at 17.1 Å (Fig. 3c). The unequal distance raises a question as to which iron ion the electrons are transferred. In natural redox proteins, physiological tunneling electron transfer can happen 6–14 Å between redox centers^28^. Therefore, we suggest that, in *Ft*AlkBG, an electron is transferred from the [Fe–4S] cluster to Fe1. Considering the close distance of 6.1 Å between Fe1 and Fe2, Fe1 may transfer an electron to Fe2. Fe1 may function as a redox center to relay electrons from [Fe–4S] in *Ft*AlkG to Fe2 in *Ft*AlkB.

## Discussion

**Fig. 4:**
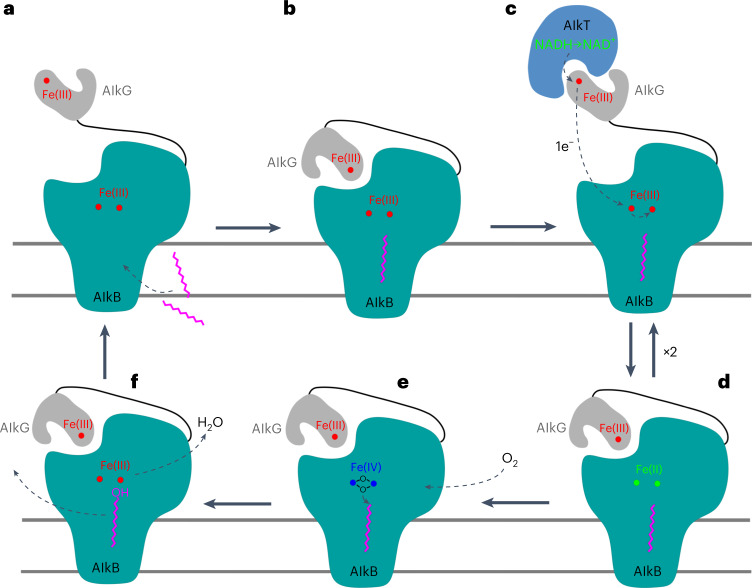
A proposed model of alkane terminal C–H functionalization. **a**, In ambient atmospheric oxygen conditions, both AlkB and AlkG are in an oxidized state with AlkG being dislodged. Fe(III)–Fe(III) is shown as red spheres. **b**, Substrate alkane can diffuse into the substrate entry tunnel in the membranes, and its terminal C–H is oriented toward to the diiron center in AlkB. **c**,**d**, When AlkT and NADH are available, AlkG shuttles two electrons, one at a time, from oxidation of NADH in AlkT to the diiron center in AlkB. Fe(III)-Fe(III) in oxidized AlkB (**c**) is reduced to Fe(II)–Fe(II) (green spheres) (**d**). **e**, Reduced diiron center activates oxygen to produce an active high-valent diiron-oxo intermediate Fe(IV)–Fe(IV) (blue spheres) that attacks the terminal C–H bond. **f**, The abstraction of hydrogen followed by addition of an OH group completes the two-electron hydroxylation process. The release of product will likely involve conformational changes in AlkB and the release of AlkG.

Alkanes are highly hydrophobic and are almost immiscible with water, but they can diffuse into hydrophobic membranes. A structural feature of the transmembrane protein AlkB is its alkane-accessible tunnel with an entrance in the transmembrane region, allowing substrate access. We expressed the protein in the absence of reductant and in the presence of supplemented ferric chloride and purified it under ambient atmospheric oxygen conditions. Our previous Mossbauer spectroscopy findings identified that the ‘as-isolated AlkB’ from *P. oleovorans* was in the Fe(III)-Fe(III) state^22^; therefore, we postulate that, in an isolated state, the iron ions in the *Ft*AlkBG structure are also in the oxidized Fe(III)-Fe(III) state (Fig. 4a), consistent with the observed long Fe–Fe at 6.1 Å (Fig. 2a). Alkanes diffuse into the membranes where they interact with the tunnel-forming hydrophobic residues that lead toward the diiron center. The shape of the tunnel means that only its terminal C–H bonds can reach the cavity adjacent to the diiron center (Figs. 2b and 4b). Binding of a substrate to the active site triggers conformational changes that induce the docking of AlkG to AlkB (Fig. 4b). When AlkT and NADH are available, AlkG will be reduced and will transfer two electrons derived from oxidation of NADH in AlkT to the diiron center in AlkB. The process requires two single electron transfer cycles from AlkT to AlkG and from AlkG to AlkB (Fig. 4c,d). The fully oxidized Fe(III)-Fe(III) diiron center is not functional because of its long Fe–Fe distance of 6.1 Å. For AlkB to be functional, its diiron site needs to be reduced to Fe(II)-Fe(II) to activate a molecular oxygen (O_2_)^22,29,30^. Binding of O_2_ to the reduced Fe(II)-Fe(II) diiron site facilitates O_2_ activation and, upon cleavage of the O–O bond, an as-yet-uncharacterized high-valent Fe–O intermediate species is created^31^. In the high-valent intermediate, the Fe–Fe distance would be substantially shorter to form the hypothesized Fe(IV)-Fe(IV)-O_2_ diamond-core intermediate structure^32^ (Fig. 4e). The diamond core is reactive and abstracts a hydrogen from the terminal C–H bond followed by oxygen rebound^33^ to form the terminal hydroxyl group. One of the two oxygen atoms is utilized for the production of the hydroxyl group during substrate oxidation, whereas the other is likely protonated by Glu271 to form a water molecule (Figs. 2a and 4f).

Biocatalysts provide environmentally benign alternatives to high-temperature and high-pressure precious metal-based catalysis for alkane C–H bond activation and functionalization^6,34^. The *Ft*AlkBG structure may provide a structural platform, in a similar way to the P450 system^35^, for creating novel biocatalysts for super-selective production of chemical feedstocks and value-added products from abundant alkanes.

## Methods

### Protein expression and purification

The gene encoding the full length of *Ft*AlkBG was synthesized and cloned into the pET16b expression vector by GenScript. The vector contains an N-terminal 10×His tag followed by a tobacco etch virus (TEV) cleavage site. Protein expression was performed in *Escherichia coli* BL21-Gold (DE3) pLysS cells (Agilent) growing in LB medium with an induction of 0.4 mM IPTG at 28 °C for 4.5 h. One milliliter of 0.1 M FeCl_3_ in 0.12 M HCl was added to every 1 L of culture 15 min before induction. Cells were harvested by centrifugation at 5,000*g* for 10 min at 4 °C, and were resuspended in lysis buffer containing 30 mM HEPES, pH 7.5, 150 mM NaCl and 2 mM MgCl_2_. Cells were lysed using an EmulsiFlex-C3 homogenizer (Avestin) at 15,000 psi. Cell lysates were cleaned by centrifuging at 25,000*g* for 25 min at 4 °C, and the supernatants were collected for pelleting membranes by ultracentrifugation at 360,000*g* for 2 h at 4 °C. The membranes were frozen in liquid nitrogen and stored at −80 °C until further use.

Membranes were resuspended in solubilization buffer (30 mM HEPES, pH 7.5, 150 mM NaCl, 20% glycerol (wt vol^−1^), 0.5 mM Tris(2-carboxyethyl)phosphine (TCEP), 8% DDM (wt vol^−1^) and Roche proteases inhibitor cocktail). After solubilization for 4 h at 4 °C, insoluble components were removed by ultracentrifugation at 360,000*g* for 30 min at 4 °C. The supernatant was applied to pre-equilibrated Ni^2+^-nitrilotriacetic acid (NTA) column (Anatrace) and washed by 8 column volumes of wash buffer (30 mM HEPES, pH 7.5, 150 mM NaCl, 20% glycerol, 0.25 mM TCEP, 50 mM imidazole, 0.05% DDM). The protein was eluted by 500 mM imidazole and digested by TEV protease overnight at 8 °C. The His tag-removed protein was clarified by ultracentrifugation at 190,000*g* for 30 min at 4 °C, desalted by Econo-Pac 10DG desalting column (Bio-Rad) and passed through the pre-equilibrated Ni^2+^-NTA column. The flow-through was concentrated to 10 mg ml^−1^ and further purified by size-exclusion chromatography using Superdex 200 Increase (GE Healthcare) in buffer containing 20 mM HEPES, pH 7.5, 100 mM NaCl, 5% glycerol, 0.25 mM TCEP and 0.01% DDM. Fractions containing the protein were pooled and concentrated to 10 mg ml^−1^ using a 50-kDa molecular cutoff concentrator (Millipore Sigma).

### Amphipol reconstitution preparation

Reconstitution of detergent-solubilized *Ft*AlkBG (10 mg ml^−1^) into amphipol nanodiscs was performed on the basis of the previously published method^37^. Purified protein in a buffer (20 mM HEPES, pH 7.5, 100 mM NaCl, 5% glycerol, 0.25 mM TCEP, 0.01% DDM (wt vol^−1^)) was mixed with amphipol PMAL-C8 (catalog no. P5008, Anatrace) in a protein:amphipol mass ratio of 1:5 (wt wt^−1^). The mixture was incubated at 4 °C for 20 h and concentrated using a 50-kDa molecular cutoff concentrator. Then, SM-2 Bio-Beads (40 mg wet; catalog no. 1523920, Bio-Rad) were added to the concentrated mixture, and the mixture was nutated at 4 °C for 3 h. Before use, Bio-Beads were soaked with buffer containing 20 mM HEPE, pH 7.6, 100 mM NaCl overnight at 4 °C. The Bio-Beads were removed by passing through a Micro Bio-Spin chromatography column. The flow-through containing the reconstituted particles was centrifuged at 20,000*g* for 1 h at 4 °C; the supernatant was used for size-exclusion chromatography through a Superdex 200 Increase column (GE Healthcare) in a buffer containing 20 mM HEPES, pH 7.6, 100 mM NaCl. The fractions containing the *Ft*AlkBG–PMAL-C8 nanodiscs were collected and concentrated to 5 mg ml^−1^ for cryo-EM analyses.

### Cryo-EM sample preparation and data collection

Three microliters of the *Ft*AlkBG–PMAL-C8 nanodiscs were applied to a glow-discharged (15 mA current for 15 s), 300-mesh *R* 0.6/1 UltrAuFoil Holey Gold grid (catalog no. Q350AR1A, Electron Microscopy Sciences). After waiting for 60 s, vitrification was performed using a Mark IV Vitrobot (Thermo Fisher Scientific) with a blotting condition of 5 s blot time, 0 blot force and 100% humidity at 6 °C.

Single-particle cryo-EM data were collected at the Laboratory for Biomolecular Structure (LBMS) facility at Brookhaven National Laboratory using a Thermo Fisher Titan Krios electron microscope (G3i) equipped with a Gatan K3 camera and a BioQuantum energy filter. With a physical pixel size of 0.666 Å (0.333 Å in super-resolution mode), a total dose of 60 e^−^ Å^−^^2^ was fractioned to 50 frames under the super-resolution mode using the Thermo Fisher data acquisition program EPU. A total of 11,757 movies were collected with an energy filter width of 15 eV throughout the data acquisition. Data collection statistics are listed in Table 1.

### Cryo-EM data processing

Dose-fractioned movies were corrected and averaged using MotionCorr2 (ref. ^38^) with a bin factor of two. Averaged movie frames were further corrected by contrast transfer function estimation using Gctf^39^. Micrographs with an estimated resolution better than 4.5 Å were selected for particle picking. Particles were initially picked and cleaned up by 2D class averaging in Relion3 (ref. ^40^). The cleaned particles were then used for training and particle picking using Kpicker^41^. A total of 2,950,051 particles were picked, extracted at 256 pixels and binned to 64 pixels with a pixel size of 2.664 Å.

We used CryoSPARC^42^ for additional 2D class averaging, which resulted in 1,738,105 selected particles. With four classes each, selected particles were used for three cycles of ab initio reconstruction followed by three-dimensional (3D) heterogenous refinements with a particle size of 64 pixels (2.664 Å). The process selected 369,194 particles, which were centered and re-extracted at 256 pixels and binned to 128 pixels with a pixel size of 1.332 Å (Extended Data Fig. 3).

Re-extracted particles were auto-refined to convergence in Relion3, followed by nonalignment 3D classification into four classes (Extended Data Fig. 3). Particles from the 3D class with the best structural feature (ɑ-helices and side chains) as visualized in Chimera^43^ were selected. These particles (13%) from two such classes were selected for contrast transfer function refinement and Bayesian polishing in Relion3, followed by 2D classification and nonuniform refinement in CryoSPARC to reach a refined reconstruction at 2.76 Å. Local resolutions were estimated using BlocRes^44^. Reconstruction statistics are listed in Table 1.

### Model building and refinement

To boost side chain features of the map, the masked and filtered cryo-EM map was sharpened using PHENIX^45^ with a *B* factor of −66 Å^2^. COOT^46^ was used to build the atomic model, including irons and a substrate dodecane. All refinements were performed in real space in PHENIX. The refined model was validated using MolProbity^47^ and the refinement statistics are listed in Table 1.

### Model visualization

The cryo-EM density map and atomic models were visualized using either PyMOL (http://www.pymol.org/) or Chimera^43^.

### Expression of AlkT

AlkT is a flavin adenine dinucleotide-dependent rubredoxin reductase that transfers electrons from NADH to *Ft*AlkBG for C–H activation and functionalization^10,12^. The gene encoding AlkT from *P. oleovorans* (*Po*AlkT) was amplified using PCR and cloned into the pETDuet expression vector. Protein expression was performed in *E. coli* BL21-Gold (DE3) pLysS cells (Agilent) growing in TB medium with an induction of 0.4 mM IPTG at 16 °C for 17 h. Cells were harvested by centrifugation at 5,000*g* for 10 min at 4 °C, and were then resuspended in lysis buffer containing 50 mM Tris-HCl, pH 8.0, 100 mM KCl, 16% glycerol, 1 mM TCEP, 2 mM K_3_PO_4_ and 2 mM MgCl_2_. Cells were lysed by passing through a French press twice at 13,000 psi. Cell lysates were cleaned up by centrifugation at 25,000*g* for 30 min at 4 °C, and the supernatants were applied to pre-equilibrated Ni^2+^-NTA resin in a buffer containing 50 mM Tris-HCl, pH 8.0, 100 mM KCl, 16% glycerol, 0.5 mM TCEP, 2 mM K_3_PO_4_ and 20 mM imidazole. After a wash with 10 column volume of wash buffer (50 mM Tris-HCl, pH 8.0, 100 mM KCl, 16% glycerol, 0.5 mM TCEP, 2 mM K_3_PO_4_, 5 mM ATP, 10 mM MgCl_2_ and 50 mM imidazole), the protein was eluted with the lysis buffer supplemented with 500 mM imidazole. The protein buffer was changed to 50 mM Tris-HCl, pH 8.0, 200 mM KCl, 16% glycerol, 2 mM K_3_PO_4_, 0.5 mM EDTA and 1 mM TCEP using a concentrator. Purified protein was frozen in liquid nitrogen and stored at −80 °C until further use.

### Enzyme activity

For the activity assay, *Ft*AlkBG was overexpressed in *E. coli* BL21 Star cells (Thermo Fisher Scientific). Cells grew in LB medium supplemented with 100 μM FeCl_3_. After growing for 14–19 h at 16 °C, cells were harvested by centrifugation at 5,000*g* for 10 min at 4 °C. Cells were resuspended in lysis buffer (30 mM Tris-HCl, pH 7.75, 150 mM NaCl, 0.2 mM PMSF, 100 μM ferrous ammonium sulfate hexahydrate and 100 μM sodium hydrosulfite) and lysed using a French press at 13,000 psi. Lysates were centrifuged at 10,000*g* for 25 min at 4 °C. Pellets were resuspended in lysis buffer without PMSF, frozen in liquid nitrogen and stored at −80 °C until further use.

The dodecane assay was performed on the basis of a modified protocol from McKenna and Coon^31,48^ using a Spark 20 M multimode microplate reader (Tecan). In a reaction volume of 100 μl, we added 20 μg *Ft*AlkBG, 10 μg *Po*AlkT and 500 μM NADH in a buffer of 50 mM Tris-HCl, pH 7.5, 100 mM NaCl. The reaction was initiated by addition of 400 μM dodecane dissolved in acetone at 24 °C. The reaction was monitored by measuring the decrease of NADH fluorescence (excitation/emission at 360/460 nm with a bandwidth of 10 nm). Consumption of NADH, that is, decreased NADH fluorescence, is proportional to dodecane hydroxylation to dodecanol. For the control experiment, we added all components except we used acetone instead of dodecane.

### Reporting summary

Further information on research design is available in the Nature Portfolio Reporting Summary linked to this article.

## Online content

Any methods, additional references, Nature Portfolio reporting summaries, source data, extended data, supplementary information, acknowledgements, peer review information; details of author contributions and competing interests; and statements of data and code availability are available at 10.1038/s41594-023-00958-0.

## Supplementary information

Reporting Summary

## Data Availability

The 3D cryo-EM density map has been deposited in the Electron Microscopy Data Bank under the accession number EMD-28890. Atomic coordinates have been deposited in the Protein Data Bank under the accession number PDB 8F6T. Source data are provided with this paper.

## Source data

Source Data Extended Data Fig. 2a Uncropped SDS–PAGE gel.
Source Data Extended Data Fig. 2b Size-exclusion chromatography curve data points.
Source Data Extended Data Fig. 4d Fourier shell correlation data points.
Source Data Extended Data Fig. 9 Activity assay data points.
