# Supplementary figures and images for: Structural basis for enzymatic terminal C–H bond functionalization of alkanes
Source: Nat Struct Mol Biol. 2023 Mar 30;30(4):521–6. doi: 10.1038/s41594-023-00958-0 (PMC10113152; doi:10.1038/s41594-023-00958-0)

## Extended Data Fig. 2a

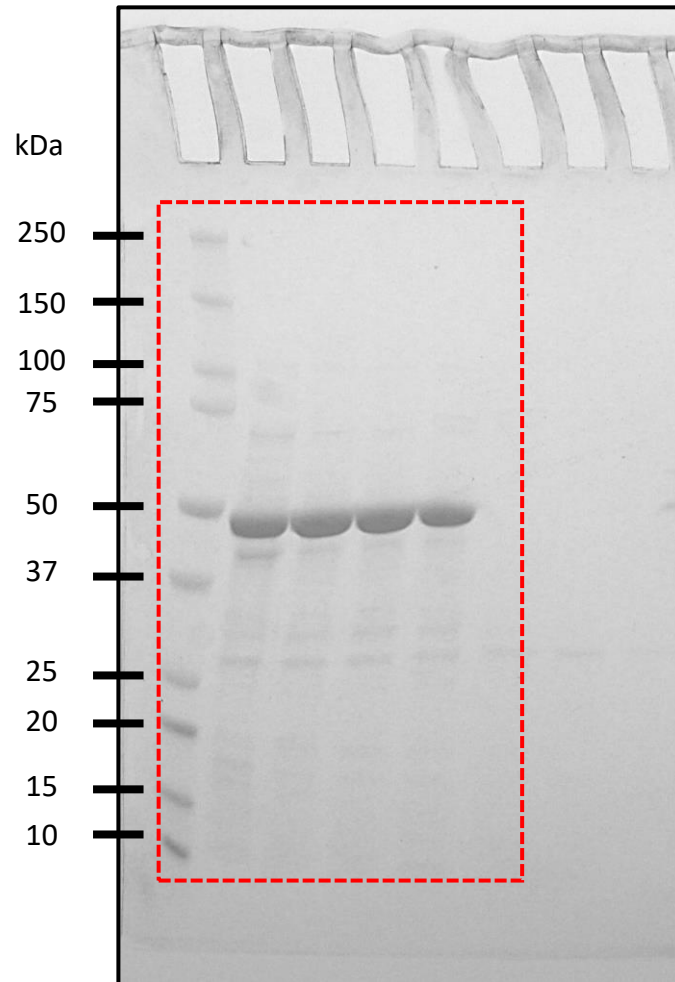

Extended Data Fig. 2a in the manuscript is marked in a dashed red rectangle.

Supplement: Source Data Extended Data Fig. 2a — Uncropped SDS–PAGE gel. [file 41594_2023_958_MOESM2_ESM.pdf]
